# Treatment of mesial temporal lobe epilepsy with amygdalohippocampal stimulation: A case series and review of the literature

**DOI:** 10.3892/etm.2013.968

**Published:** 2013-02-20

**Authors:** BAO MIN, LUAN GUOMING, ZHOU JIAN

**Affiliations:** Department of Functional Neurosurgery, Beijing Sanbo Brain Hospital, Capital Medical University, Beijing 100093, P.R. China

**Keywords:** mesial temporal lobe epilepsy, amygdalohippocampal complex, deep brain stimulation, effect

## Abstract

Deep brain stimulation (DBS) is being used with increasing frequency for the treatment of mesial temporal lobe epilepsy (MTLE). Here, we report two patients treated with amygdalohippocampal (AH)-DBS for drug-resistant temporal lobe epilepsy. Two patients with temporal lobe epilepsy were admitted to Beijing Sanbo Brain Hospital. The first patient was a 34-year-old male with a 31-year history of epileptic seizures. The second patient was a 27-year-old male with a 19-year history of drug-resistant epilepsy. The patients received a comprehensive presurgical workup and were considered unsuitable candidates for resective surgery. AH-DBS was recommended for the two patients. The last follow-up for patient 1 was 36 months after surgery and the final parameter settings were 3.6 mA, 450 μsec, 130 Hz and cycling with 60 sec on, 180 sec off. The last follow-up for patient 2 was 18 months after surgery and the final parameter settings were 2.6 mA, 450 μsec, 130 Hz and cycling with 60 sec on, 180 sec off. The patients experienced a seizure frequency reduction of 90 and 65%, respectively, with respect to the baseline. AH-DBS is a safe, micro-invasive alternative in patients with MTLE who are not candidates for resective surgery. It effectively reduces seizures without a negative effect on memory performance.

## Introduction

Mesial temporal lobe epilepsy (MTLE) is by far the most common focal epilepsy and is often associated with pharmacoresistance. The hippocampus and amygdala are commonly involved in the initial phases of electroencephalography (EEG) discharges of seizures arising from the temporal lobe. Patients with intractable seizures due to unilateral MTLE are excellent candidates for surgical treatment and resective surgery achieves a short-term cure (seizure freedom according to Engel class IA) in up to 85% of cases and long-term cure in 57–66% of cases (1). Unfortunately, up to 30% of temporal lobe epilepsy cases are unsuitable for surgery due to the bilateral nature of the disease or concerns for the risk of memory deficit, severe amnesia following the removal of the amygdalohippocampal complex (2–4) and visual field defects, as well as cognitive impairment (5,6). Thus, surgical resection is not recommended in patients with bilateral independent temporal foci or when the eloquent cortex overlaps with the presumed epileptogenic zone.

Deep brain stimulation (DBS) is being used with increasing frequency as a treatment for drug-resistant epilepsy. Various targets are approached, including seizure spread relays and direct focus stimulation (7–10). Randomized clinical trials studying open and closed-loop systems have been reported (11,12). In 2000, Velasco *et al* proposed the use of amygdalohippocampal DBS (AH-DBS) to control MTLE (13). In that study, 10 patients with diagnostic hippocampal electrodes were used for the trial of subacute hippocampal stimulation prior to temporal lobectomy. In seven of the cases, seizures stopped and interictal spikes demonstrated a significant reduction following subacute stimulation. In the subsequently published case series of chronic hippocampal stimulation, more than half of the patients experienced a seizure reduction >50% (9,14–16). Although the mechanism of action remains unclear, AH-DBS has become a selective temporal lobe epilepsy treatment.

Here, we report two patients treated with AH-DBS for drug-resistant temporal lobe epilepsy. The study was carried out with approval from the Ethics Committee of Beijing Sanbo Brain Hospital (Beijing, China) and according to the Declaration of Helsinki. Informed consent was obtained from all patients involved.

## Case reports

### Case 1

#### History

The patient was a 34-year-old male who came to Beijing Sanbo Brain Hospital with a 31-year history of epileptic seizures. The history of growth and development were normal. There was a history of febrile seizures starting at three years old, with a gradual emergence of repeated seizures. Habitual seizures were complex partial seizures with behavioral arrest and secondarily generalized tonic-clonic seizures. Seizure frequency was 1–2 times every month. The patient received pharmacotherapy with maximally tolerable doses of carbamazepine for ten years and seizure frequency was controlled to 1–2 times every year. However, the seizure frequency of the patient then increased to 2–3 times every month and the severity increased. Thus, the patient received presurgical evaluation.

#### Presurgical evaluation

A comprehensive presurgical workup was performed, including clinical history, neurological examination, video EEG (V-EEG), magnetic resonance imaging (MRI, 1.5 Tesla; Siemens, Germany), magnetoencephalography (MEG) and neuropsychological assessment. MRI scans revealed left hippocampal sclerosis. V-EEG monitoring continued for 14 days and recorded three seizures. The interictal EEG revealed epileptiform discharges in the left hemisphere; however, the ictal EEG revealed epileptiform discharges originating in the right temporal lobe. MEG revealed diffuse epileptiform discharges in the left temporal and central areas. Neuropsychological assessment revealed that the patient had moderate cognitive and memory damage. With suspected temporal lobe epilepsy and to further clarify the side of the onset zone, the patient underwent invasive monitoring with stereotactic implantation of depth and strip electrodes (covering mesial and lateral temporal lobes bilaterally). The interictal EEG of invasive monitoring revealed asynchronous spikes in the right frontotemporal and left temporal lobe and the ictal EEG revealed epileptiform discharges originating in the right hippocampus. The patient was presented at a multi-disciplinary conference and recommended for AH-DBS.

#### Surgical procedure and parameter settings

**Figure 1:**
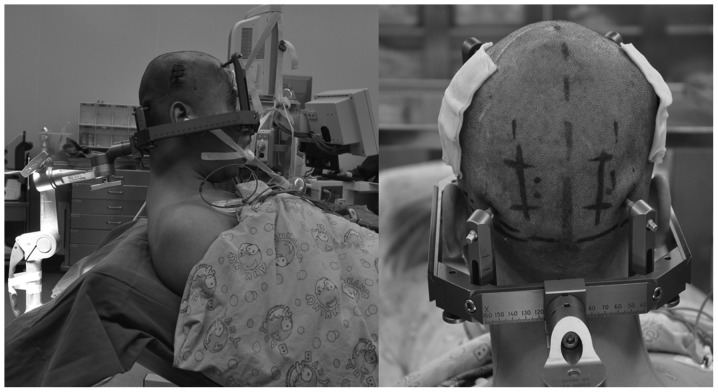
Operative incision and position.

**Figure 2:**
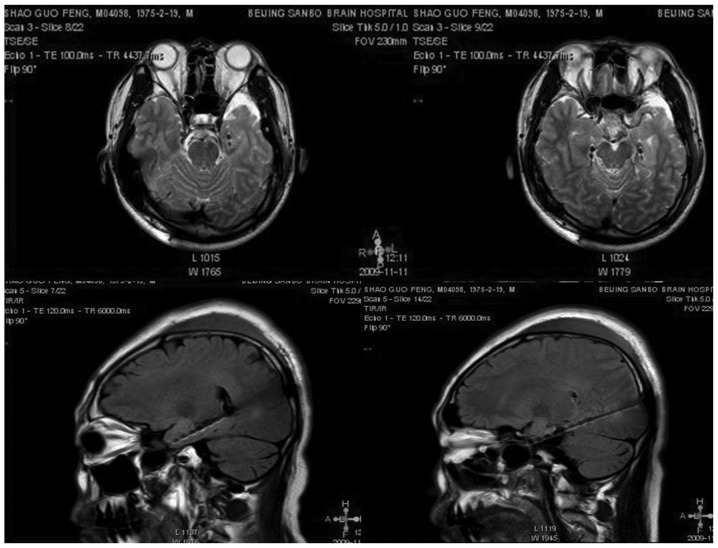
Post-surgery magnetic resonance imaging.

The patient was fixed with a stereotactic head frame (Leksell G frame, Elekta Instruments AB, Stockholm, Sweden) under local anesthesic and received an MRI scan in stereotactic conditions. The MRI data were transferred to the surgery planning system (Elekta Instruments AB). Targeting was performed by direct visualization of the amygdalohippocampal junction (Fig. 1) and the lead path through the axis of the body of the hippocampus. The patient was implanted with a quad-contact electrode (model 3146, St. Jude Medical, St. Paul, MN, USA) under the guidance of the stereotactic system to the bilateral intended target through the posterior occipital approach, under local anesthesia and in a semi-sitting position. After an X-ray was used to confirm correct target positioning, a programmable internal pulse generator (model 3716, St. Jude Medical) was implanted in a subclavicular subcutaneous pouch and connected with the electrodes by means of extension wires under general anesthesia (Fig. 2).

The generator was turned on 4 weeks after surgery. Initial stimulating parameters were as follows: current intensity, 1.5 mA; on-period, 60 sec; off-period, 180 sec; pulse width, 450 μsec and stimulating frequency, 130 Hz. In addition, the first two contacts were used as cathodes and the case box as an anode. The current intensity was gradually increased to 2.0 mA in the first month after surgery and the patient was observed for 3 months. Medication was maintained at 800 mg/day carbamazepine following surgery.

#### Long term follow-up

Follow-up and adjustments of parameters were conducted by the epileptologist, in a single blind design. The patient had parameter adjustment and machine tests once every 3 months. The seizure frequency at baseline was determined by recording the number of monthly seizures and then averaging the number of monthly seizures relative to the last 3 months before the implant. The clinical outcome was determined by comparing seizure frequency (the number of seizures/month) following AH-DBS with the baseline. The last follow-up was 36 months after surgery and the final parameter settings were 3.6 mA, 450 μsec, 130 Hz and cycling with 60 sec on, 180 sec off. The patient experienced a seizure frequency reduction of 90% in respect to the baseline. Additionally, seizure duration became shorter and the severity of attack was reduced. The patient was reported to have improved quality of life by relatives.

### Case 2

#### History

A 27-year-old male was referred for a 19-year history of drug-resistant epilepsy. The patient had experienced stereotypic seizures since childhood and the first seizure occurred aged 8 years due to fever. Seizure was characterized by a sudden tonic-clonic seizure with loss of consciousness for ∼5 min, without aura. After the first seizure, they began to repeatedly appear and occasionally secondary general tonic-clonic seizures occurred. Habitual seizures were absent. Pharmacotherapy was administered with maximally tolerable doses of valproate, carbamazepine and phenobarbital in mono- and polytherapy; however, seizure frequency was still 10–15 times every month. Due to the disappointing results of the drug treatment, the patient received presurgical evaluation.

#### Presurgical evaluation

For presurgical workup, neurological examination, MRI, V-EEG, MEG, fluorodeoxyglucose-positron emission tomography (FDG-PET) imaging and neuropsychological assessment were performed, as well as evaluation of the patient’s clinical history. MRI scans revealed bilateral hippocampal sclerosis. Scalp EEG monitoring continued for 2 days and recorded three seizures. The interictal EEG revealed asynchronous epileptiform discharges in the bilateral temporal lobe and the ictal EEG revealed diffused epileptiform discharges in the left hemisphere. MEG revealed epileptiform discharges in the bilateral temporal lobe. FDG-PET identified low metabolic activity in the left temporal lobe. Neuropsychological assessment revealed that the patient had moderate cognitive and memory damage. The patient underwent invasive monitoring with the stereotactic implantation of depth and strip electrodes. The interictal EEG of invasive monitoring revealed asynchronous spikes in the bilateral temporal lobe and the ictal EEG revealed epileptiform discharges originating in the left hippocampus and the base of the temporal lobe. The patient was also recommended for AH-DBS following the multidisciplinary conference.

#### Surgical procedure and parameter settings

The surgical procedures and parameter settings were the same as in case 1. Medication was changed to 900 mg/day oxcarbazepine following surgery.

#### Long term follow-up

The last follow-up was 18 months after surgery and the final parameter settings were 2.6 mA, 450 μsec, 130 Hz and cycling with 60 sec on, 180 sec off. The patient experienced a seizure frequency reduction of 65% in respect to the baseline. Additionally, seizure duration was shorter and the severity of attack was reduced. The patient was reported to have an improved quality of life by relatives.

## Discussion

DBS has become established as a long-term safe and effective treatment for movement disorders (17). There is a great interest in the use of DBS as an innovative treatment for drug-resistant epilepsy. A number of targets have been attempted, including the centromedian thalamic nucleus (18,19), the caudate nucleus (20), the locus coeruleus (21), the anterior thalamic nucleus (22) and the subthalamic nucleus (23). Electrical stimulation of these targets aims to activate a postulated anticonvulsant control system in the brain that restores the imbalance between excitatory and inhibitory processes that led to the epileptic seizures (24). These are classified as indirect electrical stimulation neuromodulation. However, for patients with MTLE, the treatment results of stimulation of these targets are not ideal (25).

Electrical seizure onset in the amygdala and hippocampus is the key feature of MTLE (26). The target of AH-DBS is the primary epileptogenic focus and is classified as direct stimulation neuromodulation.

Here, we reported two cases of successful AD-DBS in patients who were diagnosed with MTLE. For the first patient, the onset zone was located in the right temporal lobe, as confirmed by invasive V-EEG. However, due to left hippocampal sclerosis and the risk of memory dysfunction with resective surgery, as well as the possibility of not achieving complete seizure treatment due to the interictal epileptic discharge identified by EEG and MEG, we did not recommend resective surgery.

Similarly, patient 2 had a seizure onset zone located at the left temporal lobe and hippocampus; however, due to the bilateral hippocampal sclerosis and PET results indicating hypometabolism of the right temporal lobe, removal of the left hippocampus may have resulted in severe memory dysfunction. Therefore, this patient was also not a good candidate for resective surgery.

Prior to the use of neuromodulation, patients that were excluded as candidates for temporal lobectomy and left with no other alternative, had to suffer great physical and psychological damage. With the development of neuromodulation technology, more and more patients with drug-resistant epilepsy benefit.

AH-DBS as a treatment of MTLE reduces the seizure frequency in the majority of patients by >50%. In a number of cases the frequency is reduced by 90% and certain patients become seizure free (9,14–16,27). Several cases reported a seizure frequency lower than the baseline levels once electrical stimulation had ended and this phenomenon was attributed to residual anticonvulsive effect (28,29). The main reason for the two patients being accepted for AH-DBS was out of concern for the potential risk of memory deficit with temporal lobectomy. Memory decline following temporal lobectomy has been documented in several studies; however, no AH-DBS patient demonstrated such a decline, not even with bilateral stimulation (27). Seizure reduction occurred in all patients in our case series who accepted AH-DBS and in one patient the seizure frequency was reduced by up to 95%. Additionally, neither of the patients demonstrated a memory deficit.

Currently, there is no consensus on the most appropriate choice of stimulus parameters. Experimental evidence in animals indicates that prolonged low frequency stimulation (1 Hz applied for 10–15 min) inhibits the development and expression of amygdala-kindled seizures (30); however, this has not been confirmed in human trials. A number of researchers select low-frequency electrical stimulation (0.1–25 Hz), while others select high-frequency electrical stimulation (90–130 Hz). In the present cases, we selected the high-frequency electrical stimulation of 90 Hz.

One study considered that patients with hippocampal sclerosis require a strong stimulation (high stimulus amplitude, at ≥1 V and/or multipolar configuration) in order to decrease the seizure frequency. Furthermore, when the amplitude of the bipolar stimulation is <1 V, the seizure frequency increases (31). The impedance of case 1 was 450 (left) and 380 Ohms (right) and the set current was 3.6 mA. According to Ohm’s law (I=U/R), the voltage was ∼1.6 (left) and 1.4 V (right). The voltage in case 2 was ∼1.3 (left) and 1.1 V (right).

We identified that although the generators were not turned on in the first month after implantation, the seizure frequency reduction was observed in the two cases. Conversely, the seizure frequency increased in the first month after the generator was turned on. In one case, the seizure frequency even increased beyond the baseline and then gradually decreased with the strength of the current. The decrease of seizure frequency in the first month after implantation may be related to microlesional effects, a phenomenon that was reported by Schulze-Bonhage *et al* (32). As the microlesional zone gradually repaired, the current intensity remained at a low level (<1 V). At that time, the seizure frequency increased again until the current was gradually increased. After that, the seizure frequency decreased until it reached a steady state. Therefore, higher currents may be more effective at controling seizure attacks, so the current should be strengthened to the effective level necessary. Early control of seizures is likely to enhance the patients’ confidence in the treatment. A clear limitation of this study is the small sample size. It should be considered that only two subjects were included, leading to the possibility of selection bias.

The choice of pulse width and stimulus contacts may change the stimulus range. A larger stimulus range is considered to have a better control effect; however, it also involves greater stimulus-related discomfort, faster battery consumption and in certain cases, possible seizure increase. In addition, it remains inconclusive whether continuous stimulation or intermittent stimulation is more effective.

AH-DBS proved to be safe with no side-effects. Similar to the use of DBS in the treatment of other diseases, the most significant potential complication is hemorrhage, reported in ∼5% of patients (33). Hemorrhage usually does not require surgical treatment. Improved surgical planning, careful surgery and reduced repeated puncture help to avoid the chance of this complication. In addition, skin erosions and infection are the other main complications of the placement of stimulation devices, particularly in children or thin patients (27). Although all precautions are taken to avoid skin erosion and infection, it is difficult to completely avoid them. It may be of value to improve the stimulation system design to make it smaller and more compatible. In our cases, none of these side-effects were observed.

One study demonstrated that the reduction of seizure frequency following hippocampal stimulation is <50% (34). Other studies demonstrated prolonged seizure control in patients who underwent invasive recording with conventional electrodes. A number of studies support the hypothesis that actual stimulation is not necessary to achieve efficacy and claim that efficacy is based on the electrode microthalamotomy effect that is provoked by the insertion of the electrodes (35,36). The indications and results are not yet fully validated and the therapeutic objective should remain palliative. In addition, the mechanism of action of DBS in reducing seizures remains unclear; however, it should be recognized that, at this point in time, DBS is a promising treatment option for a subgroup of carefully selected patients with MTLE who are not suitable candidates for resective surgery. Furthermore, more cases and control studies are required to refine the patient selection criteria, anatomical targets and ideal stimulation parameters. With more extensive trials, AH-DBS in MTLE is likely to become a valuable alternative.

AH-DBS is a safe, micro-invasive alternative in patients with MTLE who are not suitable candidates for resective surgery. It effectively reduces seizures without a negative effect on memory performance. Currently, the most appropriate choice of stimulus parameters remains unclear. A larger sample size and well-designed randomized control studies are required to elucidate the impact of this treatment.
